# The role of sex hormones in neurological and functional recovery following spinal cord injury in females: a systematic review of the literature

**DOI:** 10.1038/s41393-026-01201-5

**Published:** 2026-04-11

**Authors:** Ema Vidakovic, Elisabeth Bamberg, Björn Zörner, Kathleen Ostheim, Beatrice Minder, Sue Bertschy, Juergen Pannek, Petra Stute, Marija Glisic

**Affiliations:** 1 Swiss Paraplegic Research, Nottwil, Switzerland; 2 Faculty of Health Sciences and Medicine, University of Lucerne, Lucerne, Switzerland; 3 Institute of Social and Preventive Medicine, University of Bern, Bern, Switzerland; 4 Swiss Paraplegic Center, Nottwil, Switzerland; 5 Public Health & Primary Care Library, University Library of Bern, University of Bern, Bern, Switzerland; 6 Department of Obstetrics and Gynecology, University Clinic Inselspital Bern, Bern, Switzerland

**Keywords:** Predictive markers, Outcomes research

## Abstract

**Study design:**

Systematic review.

**Objective:**

To synthesize human and animal evidence on the potential neuroprotective effects of sex hormones in women with spinal cord injury (SCI) and in female animal models, with the aim of comparing findings across experimental and clinical studies and identifying gaps in clinical research.

**Methods:**

EMBASE, Medline, Cochrane CENTRAL, and Web of Science were searched from inception to August 22, 2025, without language restrictions. Two reviewers independently performed study screening, data extraction, and risk of bias assessment.

**Results:**

Of 2832 screened records, 26 studies were included (23 preclinical and 3 clinical). Human evidence was scarce and primarily addressed functional recovery after acute SCI, as well as cardiovascular risk factors and bone outcomes in small cohorts of pre- and postmenopausal women with chronic SCI. Preclinical studies consistently demonstrated broad neuroprotective effects of estradiol across multiple outcomes. Inhibition of estrogen synthesis with letrozole was associated with impaired motor recovery, while the presence of intact ovaries was linked to improved sensory outcomes, supporting a protective role of endogenous estrogens. Evidence for progesterone and dehydroepiandrosterone (DHEA) was mixed, although DHEA showed promising effects in two of three studies, including improved motor and sensory recovery and tissue preservation.

**Conclusion:**

This review highlights the therapeutic potential of sex hormones in SCI recovery while also revealing substantial gaps in human research. Future studies should investigate endogenous hormone profiles and hormone-based interventions during the acute and subacute phases of SCI to guide therapeutic development and bridge the gap between animal models and clinical practice.

## Introduction

Traumatic spinal cord injury (SCI) results from a sudden external force such as a traffic collision, fall, sports injury, or act of violence that causes direct damage to the spinal cord, leading to partial or complete loss of motor, sensory, and autonomic functions [1]. Traumatic SCIs are notably more prevalent in males, accounting for approximately 80% of cases, compared to around 20% in females [2, 3]. The age distribution follows a bimodal pattern, with a first peak between 15 and 29 years and a second, smaller but steadily increasing peak among individuals older than 50 years [3–5]. Men remain at higher risk across all age groups, however, the male-to-female ratio narrows substantially after the age of 54, indicating a relatively higher incidence of SCI among older women [3–5].

Despite these changes in epidemiological patterns, women remain underrepresented in clinical SCI research [6, 7]. In contrast, more than 60% of preclinical SCI models use female animals [8]. This imbalance complicates the translation of experimental findings into clinical practice and limits our understanding of sex-specific biological mechanisms of recovery. Addressing this gap is particularly important given growing evidence that recovery trajectories after SCI may differ between women and men and may be influenced by sex hormones.

Recovery following SCI varies considerably even among individuals with similar initial clinical presentation [9, 10]. While most functional gains occur within the first three months, incremental improvements may continue for up to 18 months post-injury [11]. Key factors influencing neurological recovery include age, injury level, and severity [10, 12, 13]. Further, experimental studies in animal models of SCI have consistently shown sex-based differences in recovery favoring females [14, 15]. While the underlying mechanisms remain incompletely understood, accumulating evidence points to a potential role of sex hormones, including estrogen, progesterone, dehydroepiandrosterone (DHEA), and testosterone [16, 17]. These sex hormones modulate cellular signaling and metabolism and may shape sex-specific recovery trajectories after SCI [18]. Experimental studies suggest that progesterone, estrogens, DHEA, and testosterone exert neuroprotective effects through mechanisms such as attenuation of inflammation and edema, reduction of oxidative stress and excitotoxicity, preservation of mitochondrial function, promotion of neuronal survival, and enhancement of neuroplasticity [17, 19, 20]. These pleiotropic effects provide a strong biological rationale for sex-specific recovery patterns and highlight the potential importance of hormonal status in shaping outcomes after SCI.

In humans, however, evidence for sex differences in recovery after SCI remains limited and inconsistent. Some clinical studies suggest that women may experience greater improvements in motor function and independence [21, 22], whereas others report an increase in improvement in men, or no differences between sexes in recovery after the injury [23–25]. These inconsistencies may reflect differences in injury type, age at injury, or intrinsic biological responses. For example, women tend to sustain SCI at older ages which could potentially complicate recovery, yet these injuries may stem from less forceful events (e.g., falls vs. traffic accidents), which could influence recovery trajectories [22, 26]. Clinical evidence remains difficult to interpret due to underrepresentation of women in SCI research and insufficiently powered studies to explore sex differences in a clinical setting [6]. Given these challenges, preclinical studies remain a critical source of mechanistic insight. However, existing reviews have typically examined sex hormones across multiple forms of central nervous system injury or have combined findings from male and female SCI models, potentially obscuring injury-specific and sex-specific biological mechanisms [17, 27, 28].

The aim of the present systematic review is to evaluate the neuroprotective effects of endogenous and exogenous sex hormones after SCI, with a specific focus on women and female animal models. By integrating evidence from both clinical and preclinical studies, this review aims to facilitate meaningful comparisons between experimental and human findings, identify critical gaps in the existing literature, and inform the design of future clinical studies, ultimately supporting the development of more personalized and sex-sensitive approaches to SCI management.

## Methods

### Data source and search strategy

This systematic literature review was conducted in accordance with an established guideline for systematic reviews in medical research [29], which is aligned with the Cochrane Handbook for Systematic Reviews of Interventions [30]. The study protocol was registered in PROSPERO prior to conducting the review (CRD42023445073). The original protocol was designed to include both individuals with SCI and traumatic brain injury; however, given the distinct pathophysiology and scope of the evidence, the findings will be reported in separate scientific publications. The review reporting follows the PRISMA (Preferred Reporting Items for Systematic Reviews and Meta-Analyses) guidelines [31].

We searched the MEDLINE (Ovid), EMBASE.com, Web of Science (Core collection), Cochrane CENTRAL from inception until 22.08.2025. The search strategy (in **Appendix 1**) was created by an experienced librarian. Search terms used related to the population (i.e., Spinal Cord Injury). Regarding exposure, the search included sex hormones (estrogen, testosterone, dehydroepiandrosterone, progesterone, etc.) and female-specific conditions (such as menstrual cycle dysregulation).

### Study selection and eligibility criteria

Studies were included if they met the following criteria: (1) were conducted in women with SCI or in female animal models of SCI; (2) were designed as cohort, case-control, cross-sectional studies or randomized controlled trials, pre-post studies, or experimental pre-clinical studies; (3) assessed the association between blood sex hormone levels/or sex-hormones or their derivates effectiveness and rehabilitation outcomes (e.g., cognitive function, neurological recovery, quality of life etc.). In addition, studies that explored proxies of hormonal status were also included, such as the following female-health states and conditions (e.g., pregnancy and menopausal status at the time of injury, menstrual disruption following injury). Letters to the editor, reviews, commentaries, case studies, conference abstracts, non-peer-reviewed studies, or studies conducted in men and women, and male and female animal models without stratified analyses including females were not eligible for inclusion.

### Data extraction

Two independent reviewers screened titles, abstracts, and full-texts using EndNote in accordance with the predefined study selection criteria. Any disagreements were resolved by consultation with a senior reviewer, who made the final decision regarding study inclusion. Discrepancies most commonly arose when studies included both male and female SCI models; in such cases, inclusion was determined by consensus, for example when sex-disaggregated results were reported. Data on authorship, year of publication, country, study design, population characteristics, age, injury characteristics, cause of injury, time since injury, exposures, and reported outcomes were extracted using standardized forms in Microsoft Word.

### Risk of bias assessment

The assessment of risk of bias in human observational and intervention studies was conducted using the Quality Assessment Tool developed by the National Heart, Lung, and Blood Institute (NHLBI) [32, 33]. This tool primarily evaluates a study’s internal validity, identifying potential methodological limitations or implementation concerns. Specifically, it examines sources of bias (e.g., patient selection), confounding factors, statistical power, and the strength of causal relationships between interventions and outcomes. Two independent reviewers assessed the risk of bias by applying a checklist and selecting from the following responses for each item: “yes”, “no”, or “cannot determine (CD)/not reported (NR)/not applicable (NA)”.

For animal studies, the SYRCLE Risk of Bias (RoB) tool, a modified version of the Cochrane RoB tool, was used [34]. This adaptation accounts for bias specific to animal intervention research. The tool comprises ten criteria covering selection bias, performance bias, detection bias, attrition bias, reporting bias, and other sources of bias. Two reviewers independently categorized responses as (1) “yes” for a low risk of bias, (2) “no” for a high risk of bias, or (3) “unclear” when there was insufficient information to determine the risk. Any disagreements in the assessments were resolved through discussion, with a third reviewer consulted if necessary.

### Studies synthesis

Due to significant clinical heterogeneity among the included studies reflected in differences in study populations, designs, and outcomes of interest, a meta-analysis was not appropriate. Instead, we present a narrative synthesis of the evidence, distinguishing between human and animal studies, as well as categorizing them based on exposures or interventions used across the studies including different hormonal preparations or exposures.

## Results

**Fig. 1:**
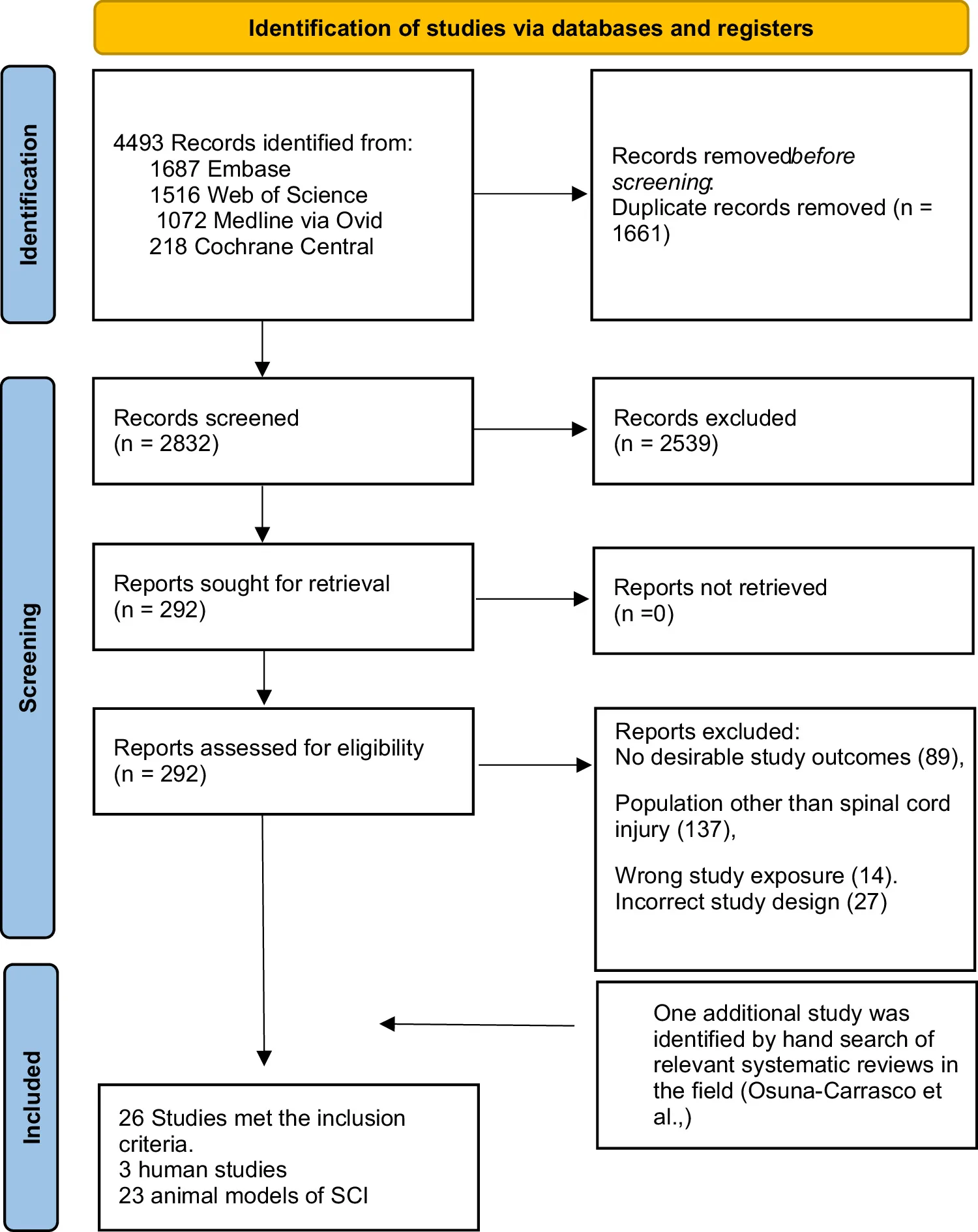
Flowchart of included studies. The search strategy included both conditions; spinal cord injury and traumatic brain injury (see Prospero protocol for details). Current review focuses only on SCI, therefore, all human and animal studies focusing on traumatic brain injury have been removed at the level of full text screening (and will be addressed in a separate publication). Source: Page MJ, et al. BMJ 2021;372:n71. https://doi.org/10.1136/bmj.n71. This work is licensed under CC BY 4.0. To view a copy of this license, visit https://creativecommons.org/licenses/by/4.0/.

The search strategy yielded 2832 potentially relevant citations. Out of these, 292 full-text articles were retrieved for further evaluation. Following this, 267 articles were excluded for the following reasons: no desirable study outcomes, non-SCI study population or inappropriate study design. One study was identified through hand search and overall, 23 animal and 3 human studies met the inclusion criteria and were included in the current systematic review, Fig. 1.

### Human evidence

Three human studies were observational and explored the association between serum levels of androgen hormones and functional recovery [35], menopausal status and intermediate cardiometabolic risk factors [36], and bone integrity [37], Table 1. The risk of bias assessment (Supplemental Table 1) revealed methodological concerns related to the definition of study populations, absence of power analyses, and a lack of longitudinal assessments. In a longitudinal study of high methodological quality, men and women with traumatic and non-traumatic SCI admitted to initial inpatient rehabilitation, low testosterone levels during the acute phase were associated with slower functional improvement (as measured by SCIM scores) and lower total functional gains in men. In women, no significant association was observed; however, only 15 women were included in these analyses, limiting the interpretability of the results [35]. In a cross-sectional study of low methodological quality, the cardiometabolic risk profiles of eleven premenopausal and six postmenopausal women with motor-complete cervical or thoracic SCI were compared. Postmenopausal women had higher mean levels of total cholesterol, triglycerides, and LDL-C compared to their premenopausal counterparts. However, only LDL-C levels in the postmenopausal group exceeded recommended values [36]. Another study of moderate methodological quality examined trabecular bone structure in the knees of pre- and postmenopausal women with SCI, as well as in women without SCI. All women with SCI had chronic cervical or thoracic injuries. Compared to ambulatory women, those with SCI showed significant deterioration in trabecular bone. Specifically, the SCI groups had 19–26% fewer and 6% thinner trabeculae, which were spaced 40–62% further apart, resulting in a 22–33% reduction in trabecular bone volume (in the tibia and femur, respectively). Measures of trabecular bone microarchitecture did not significantly differ between pre- and postmenopausal women with SCI [37].

**Table 1:** General characteristics of human studies.

Author, year of publication	Country	Study design	Population	No. of women	Injury characteristics (level and AIS score) for SCI	Cause of injury	Time since injury	Age (mean, SD)	Exposure	Reported outcomes	The main findings
Seijas et al. [35]	Switzerland	Longitudinal	Individuals ≥16 who have completed their initial rehabilitation after a newly diagnosed SCI	15	Acute cervical and thoracic, complete and incomplete SCI	Traumatic and non-traumatic	Measurements are performed at up to four occasions: one month (T1), three months (T2), and six months (T3) after SCI diagnosis during the first inpatient rehabilitation episode and at discharge (T4)	51 (IQR: 36-64)	Serum levels of androgen hormones	functioning metrics (Spinal Cord Independence Measure version III (SCIM-III) and serum androgen levels (total testosterone [TT], free testosterone [FT], sex hormone-binding globulin, dehydroepiandrosterone [DHEA], and DHEA sulfate [DHEAS])	All results for females were null
Hosier et al. [36]	USA	Cross-sectional	Pre- and postmenopausal women with SCI	17 (11 premenopausal and 6 postmenopausal)	Chronic cervical and thoracic, complete and incomplete SCI	NR	Chronic injury, time since injury not reported	Premenopausal: 32.4 y ± 10.0 Postmenopausal: 56.0 y ± 9.4	Menopausal status, self-reported through questionnaire	Framingham risk score, fasting serum total cholesterol (TC), high-density lipoprotein cholesterol (HDL-C), triglycerides (TG), and hemoglobin A1C (Hb1Ac); calculated low-density lipoprotein (LDL-C)	A significantly larger proportion of the group of postmenopausal women had higher TC (mean = 191.6 and 158.3 mg/dL; P = .05), TG (mean = 101.8 and 55.7 mg/dL; P = .01), and LDL-C). No statistically significant differences were observed for other cardiovascular risk markers nor Framingham risk score
Slade et al. [37]	USA	Case-control	Pre- and postmenopausal women with SCI and pre-and postmenopausal women without SCI	19 women with SCI (6 postmenopausal and 13 premenopausal) and 17 women without SCI (9 postmenopausal and 8 premenopausal)	Chronic cervical and thoracic injury	NR	Chronic SCI (>= 2 years post injury)	Women without SCI premenopausal: 44.90 +/− 2.90, Women without SCI postmenopausal: 56.8 +/− 4.06, Women with SCI premenopausal: 42.6 +/− 4.66, Women with SCI postmenopausal: 54.5 +/− 7.7, SCIPREY: 23.0 +/− 2.55	Menopausal status in women with SCI, and comparison among women with and without SCI	Bone integrity (degree of deterioration) of the Trabecular bone: Measures of trabecular bone volume, trabecular number, trabecular spacing, and trabecular thickness in the distal femur and proximal tibia.	The structure in women with SCI had deteriorated more ultrastructure than bone in postmenopausal women without SCI There were no significant differences in any measure of trabecular bone microarchitecture among the three groups of spinal cord injured women.

### Animal evidence

**Fig. 2:**
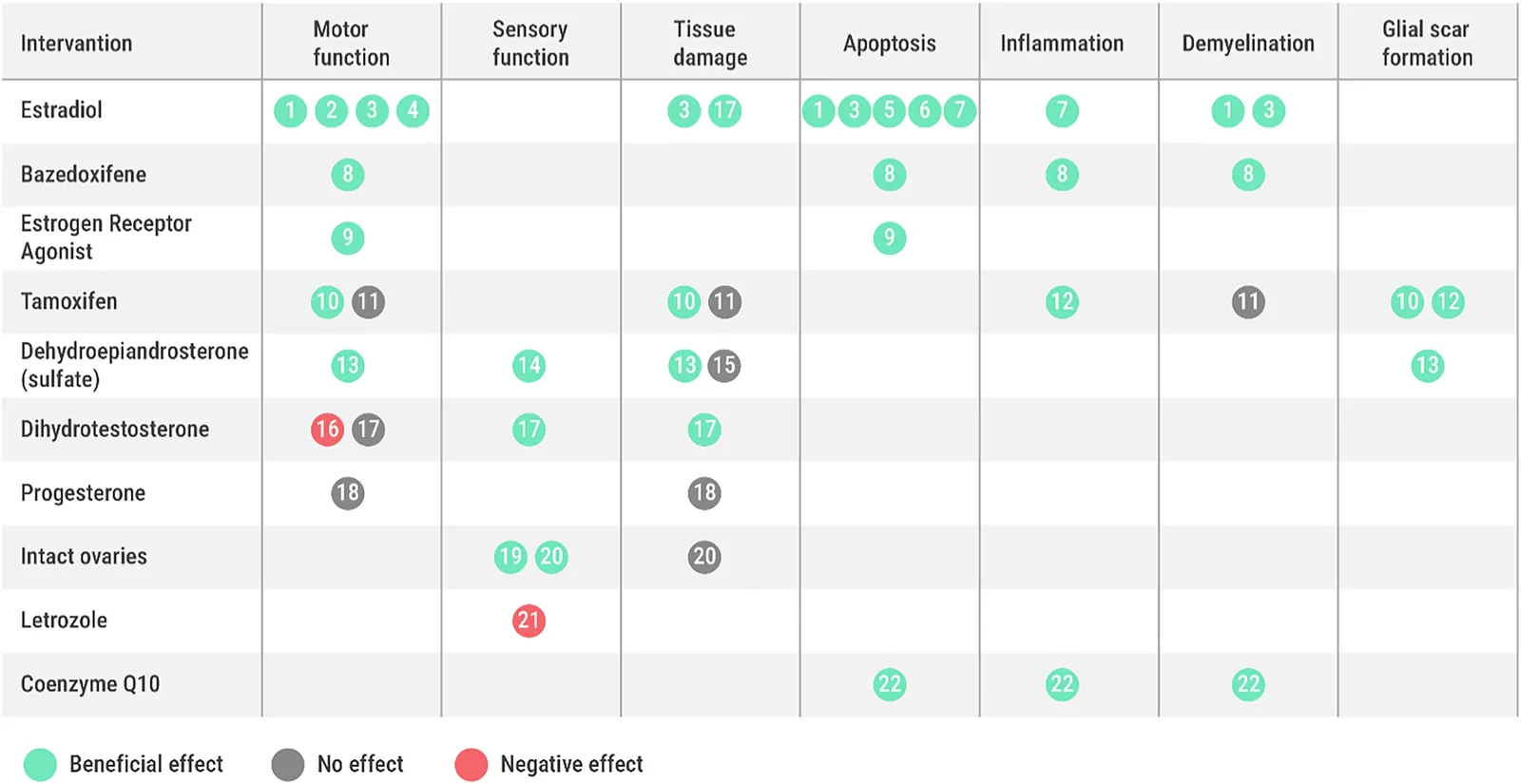
Summary of the findings from animal studies. Summary of findings from animal studies categorized by sex hormone intervention and grouped according to study outcomes. Each circle represents a single study (numbered 1 to 22). Green circles indicate a beneficial effect or improvement in the outcomes studied; grey circles indicate no effect or no improvement; red circles indicate a negative effect. Studies included in the summary: 1: Swartz et al. [39]; 2: Chaovipoch et al. [38]; 3: Lin et al. [40]; 4: Mosquera et al. [46]; 5: Li et al. [41]; 6: Rong et al. [42]; 7: Li et al. [43]; 8: Kim et al. [44]; 9: Cheng et al. [45]; 10: Colon et al. [47]; 11: Pukos et al. [49]; 12: Osuna-Carrasco et al. [48]; 13: Fiore et al. 2003; 14: Mure et al. [52]; 15: Guth et al. [53]; 16: Hauben et al. [15]; 17: Sengelaub et al. [54]; 18: Fee et al. [55]; 19: Doperalski et al. [57]; 20: Hubscher et al. [58]; 21: Ghorbanpoor et al. [59]; 22: Hassanzadeh et al. [56]; 23: Colón-Mercado, 2024.

The most used animal models in studies were Sprague–Dawley rats (N = 15, 68.18%), followed by Wistar rats (N = 3, 13.64%), and CD-1 (N = 2, 9.09%) and C57BL/6 J (N = 2, 9.09%) mice. Contusion injury was the most common injury type (N = 13, 59.09%), followed by compression (N = 7, 31.82%), and electrolytic lesions (N = 1, 4.55%) and hemisection (N = 1, 4.55%). Most of the models had intact gonads (N = 13, 59.09%), while the rest used ovariectomized models (N = 9, 40.91%). Twelve studies (54.55%) explored the role of estrogen, estrogen-like compounds or selective estrogen receptor modulators on neurological recovery post-injury. Five studies (22.73%) explored the role of androgen compounds on recovery (DHEA, DHEA(S) and DHT). Another five studies (22.73%) explored the neuroprotective effects of progesterone, coenzyme Q-10, letrozole, and ovariectomy. Table 2 provides an overview of the basic characteristics of included animal studies and Fig. 2 summarizes the most relevant results. Risk of bias assessment (Supplemental Table 2) has shown that all studies (N = 23, 100%) had a high risk of bias due to lack of allocation concealment, random housing and blinding of study personnel, while most (N = 19, 82.6%) had a high risk of bias due to not balancing baseline characteristics of animals and lack of random outcome assessment. Most studies had a low risk of bias in the following categories: selective outcome reporting (N = 21, 91.3%), other sources of bias (N = 20, 86.9%), incomplete outcome data (N = 19, 82.6%) and blinding of outcome assessors (N = 17, 73.9%).

**Table 2:** Summary of the most important study characteristics of animal studies.

	Author, publication year	Animal model						Intervention				Outcomes
		Animal type, breed, weight	Age	No. included animals	Reproductive status	Injury type	Injury level	Groups	Administration route	Dosage	Timing and duration of treatment	
**Estrogen, estrogen like compounds and selective estrogen modulators**	Chaovipoch et al. [38]	Rats, Sprague-Dawley, 200–250 g, 350–400 g	Adult (2 months) or middle age (1 year old)	130 (65 adult/premenopausal and 65 middle-aged/postmenopausal)	Ovariectomized, 1 week pre-SCI	Compression	T8-T9	8 groups: Pre-menopausal (2-month-old) 1. LAM 2. SCI + cycling 3. SCI + OVX + vehicle 4. SCI + OVX + E2 Post-menopausal (1-year-old) 5. LAM 6. SCI + non-OVX 7. SCI + OVX + vehicle 8. SCI + OVX + E2	Silastic capsule subcutaneously	180 ug/mL of 17β-estradiol	Pre/post-SCI; 1^st^ capsule immediately after ovariectomy, 2^nd^ capsule on 14 DPI (for rats surviving for 21 days)	Motor function Tissue damage Apoptosis Demyelination Sensory function
	Swartz et al. [39]	Rats, Sprague-Dawley, 192-268 g	Young adult (on average 8 weeks old)	18-24	Ovariectomy, 14 days pre-SCI	Contusion	T10	3 groups 1. OVX vehicle 2. OVX low-dose estrogen 3. OVX high-dose estrogen	Subcutaneous capsule implant	Low estradiol 180 µg/mL High estradiol 1 mg/mL	Pre/post SCI; first implants 7 days pre-SCI, second implants at 7 DPI	Motor function Tissue damage
	Lin et al. [40]	Rats, Sprague-Dawley, 200-220 g	Adult	120	Ovariectomy, 7 days pre-SCI	Compression	T9	4 groups^(f)^: 1. LAM + vehicle 2. LAM + E2 3. SCI + vehicle 4. SCI + E2	Intramuscular injection	Estradiol benzoate 0.5 mg/kg	Pre-/post-SCI; every second day since ovariectomy until sacrifice	Motor function Tissue damage Apoptosis
	Li et al. [41]	Rats, Wistar, 200-250 g	No mention	65	Ovariectomized, 2 weeks pre-SCI	Compression	T10	3 groups: 1. sham-operated 2. estrogen 3. vehicle	Intraperitoneal injection	estrogen (benovocylin 1 mg/kg)	Post-SCI; on the 4^th^ and 7^th^ days every week	Apoptosis
	Rong et al. [42]	Rats, Sprague-Dawley, 200-220 g	adult	55	Ovariectomized, 7 days pre-SCI	Contusion	T9-T10 ^(a)^	3 groups: 1. LAM + vehicle 2. SCI + vehicle 3. E2-treated	Vein injection in the tail	4.0 mg/kg E2	Post-SCI; 30 min post injury	Apoptosis
	**Li et al**. [43]	Rats, Sprague-Dawley, 200-220 g	4 weeks	48	Intact ovaries	Contusion	T10	4 groups: 1. LAM + vehicle 2. SCI + vehicle 3. DEX 4. DEX + estradiol benzoate	Intramuscular injection at the lateral thigh muscle of the forelimb	Dexamethasone (0.2 mg/kg) Estradiol benzoate (0.04 mg/kg)	Post-SCI; at 30 minutes, 1,2, 3, 4, and 5 DPI	Motor function Inflammation Apoptosis
	Kim et al. [44]	Rats, Sprague-Dawley, 180-200 g,	8 week-old	45	Intact ovaries	Compression	T9, T11	3 groups: 1. LAM + vehicle 2. SCI + vehicle 3. SCI + BZA	Intraperitoneal injection	Initial bolus of 1 mg/kg BZA pre-SCI 3 mg/kg postoperatively every 24 h for 7 days	Pre-/post-SCI; 1 h prior to the injury, every 24 h postoperatively	Motor function Tissue damage Apoptosis Inflammation Demyelination
	Cheng et al. [45]	Mice, C57BL/6 J, 22–25 g	Adult (10-12 weeks old)	40	Intact ovaries	Compression	T7–T9	5 groups: 1. Vehicle (olive oil) 2. G-1 3. E2 4. G-1 + G-15 5. E2 + G-15	Intraperitoneal injection	G-1 = 1 μg/mice daily E2 = 1 μg/mice daily G-15 = 5 μg/mice daily	Post-SCI; immediately and up to 2 weeks	Motor function Tissue damage Apoptosis Inflammation Demyelination
	Mosquera et al. [46]	Rats, Sprague-Dawley, 250 g	adult	55^(**b**)^	Ovariectomized, 7 days pre-SCI	Contusion	T9-T10	3 groups: 1. LAM + empty silastic implants 2. Estradiol benzoate 3. TAM	Subcutaneously in the back of the neck in the midscapular region:Silastic capsules: 17-β Estradiol 3-benzoate and MPP dihydrochloride Pellets: TAM	17-β Estradiol 3-benzoate 3 mg TAM 15 mg (release throughout 30 days)	Pre- and post-SCI; immediately after ovariectomy	Motor function Demyelination
	**Colon et al**. [47]	Rats, Sprague-Dawley, ~230 g	adult	126	Intact ovaries	Contusion	T9-T10	6 groups: 1. Sham PLB 2. Sham TAM 3. SCI PLB t = 0 + 24 h 4. SCI TAM t = 0 h 5. SCI PLB t = 24 h 6. SCI TAM t = 24 h	Subcutaneous implantation in the mid-scapular region	TAM pellets 15 mg	Post-SCI; immediately or 24 h post-SCI	Motor function Demyelination Apoptosis Glial scar formation
	Osuna-Carrasco et al. [48]	Rats, Sprague-Dawley, 250-300 g	Young adult	24	Intact ovaries	Contusion/transection	T13-L1	4 groups: 1. Untreated 2. TAM 3. Exercise* 4. TAM + exercise* *Exercise/locomotive training: group began training 5 days pre-SCI on a treadmill and through a passageway tunnel	Intraperitoneal injection	Tamoxifen 1 mg/kg	Post-SCI; immediately after, 24 and 48 h post injury	Functional recovery Tissue damage
	**Pukos et al**. [49]	Mice, C57BL/6 J (WT)	12-week-old	19	Intact ovaries	Contusion	T9	2 groups: 1. TAM 2. Vehicle	Oral gavage	Tamoxifen 300 mg/kg	Post-SCI; from 19 to 22 DPI	Motor function Tissue damage Apoptosis Demyelination Glial scar formation
	**Colón-Mercado** [50]	Rats, Sprague-Dawley	64–80 days old (246 ± 3 g)	34	Intact ovaries	moderate contusion to the spinal cord (12.5 mm, 10 g) using the NYU/MASCIS impactor	T9–T10	4 groups (n = 34): 1. Sham + PLB (laminectomy) 2. Sham + TAM (laminectomy) 3. SCI + PLB SCI + TAM	Subcutaneous implantation in the mid-scapular region	Tamoxifen pellets 15 mg designed for continuous drug delivery of for 21 days at a rate of 0.71 mg/d	Post-SCI – immediately	Mechanical allodynia at 21, 28, and 35 DPI with 15 g, monofilament as a baseline with the battery of the monofilaments selectedfor this paradigm was 4, 6, 8, 10, 15, 26, 60, 100, and 180 g
**Androgen hormones**	**Fiore et al**. [51]	Mice, CD-1	Adult (5-6 month old)	27	Intact ovaries	Contusion	T8	3 groups: 1. uninjured 2. SCI + vehicle 3. SCI + DHEA	Patch, intraperitoneal injections, and orally in drinking water	- Patch: 10^–10^ M DHEA - Injections: DHEA 10^–6^ M or 0.02 mg/kg/day - Drinking water: 10^–6^ M	Post – SCI - Patch: immediately after SCI (applied to the spinal cord before closure of the wound) - Injections: twice a day for the first 12 days - In drinking water up to 42 DPI	Motor function Tissue damage Demyelination Glial scar formation
	Mure et al. [52]	Mice, CD-1, mean weight 35 g	6 month old	54 or 60^(c)^	Intact ovaries	Contusion	T8	Group 1: *Behavioural assessments of bladder control and motor function recovery* 1. SCI + Vehicle) 2. SCI + DHEA Group 2: *Establishing the correlation between morphometric values and injury-induced bladder dysfunction and its pharmacological treatment with DHEA* 1. SCI + vehicle 2. SCI + DHEA 3. Sham + vehicle 4. Sham + DHEA	Patch, subcutaneous injections, and orally in drinking water	- Patch: 10^–10^ M DHEA - Injections: with DHEA 10^–6^M or 0.02 mg/kg/day - Drinking water: 10^–6^ M	Post–SCI - Patch: immediately after SCI (applied to the spinal cord before closure of the wound) - Injections: twice a day for the first 12 days - In drinking water up to 42 DPI	Motor function Sensory function Tissue damage Demyelination
	Guth et al. [53]	Rats, Sprague^–^Dawley, 170^–^200 g	No mention	54	Intact ovaries	Compression	T8	8 groups: 1. Control 2. IM 3. LPS 4. DHEAS 5. PREG 6. LPS/IM 7. DHEAS/LPS/IM 8. PREG/LPS/IM	Pellets subcutaneously (PREG, DHEAS); injections interperitonaeally (LPS, IM)	Pellets: 50 mg PREG 50 mg DHEAS Injections: 0.2 mg IM daily 0.2 mg LPS daily	Post^–^SCI; immediately	Motor function Tissue damage
	Sengelaub et al. [54]	Rats, Sprague^–^Dawley, 259.36 ± 4.6 g	~12 weeks old	47	Intact ovaries	Contusion, severe, NYU impactor (10 g dropped from 25 mm)	T9	5 groups: 1. Sham 2. SCI + empty capsules 3. SCI + DHT 4. SCI + E2 5. SCI + DHT + E2	Silastic capsules, subcutaneously	DHT levels in the normal physiological range^(i)^ E2 serum levels similar to the average peak levels in intact cycling females in the proestrous cycle	Post^–^SCI; immediately	Motor function Sensory function Demyelination Tissue damage
	Hauben et al. [15]	Rats and mice, Sprague^–^Dawley rats (200^–^250 g)	Adult 10 ± 12^–^week^–^old rats	10	Intact ovaries	Contusion	T8	2 groups: 1. DHT 2. Vehicle	Pellet, subcutaneously, neck position	Pellet DHT 100 mg	Pre^–^SCI; 10 days before SCI	Motor function
**Other interventions**	Fee et al. [55]	Rats, Sprague-Dawley, 192-268 g	6-8 week-old adult	38	Intact ovaries	Contusion	T10	a) 3 groups in 5-Day supplementation arm: 1. vehicle 2. 4 mg/kg PG 3. 8 mg/kg PG b) 3 groups in 14-Day supplementation arm: 4. Vehicle 5. 8 mg/kg PG 6. 16 mg/kg PG	No mention	a) 5-Day supplementation arm: 4 or 8 mg/kg PG b) 14-Day supplementation arm: 8 or 16 mg/kg PG	Post-SCI a) 5-Day supplementation arm: 30 min, 6 h, and once daily for 5 days b) 14-Day supplementation arm: 30 min, 6 h, and once daily for 14 days post-injury	Motor function Tissue damage Demyelination
	Hassanzadeh et al. [56]	Rats, Wistar, 200-250 g	Adult	30	Ovariectomized, 3 weeks pre-SCI	Compression	T10	5 groups: 1. OVX 2. OVX + SCI 3. OVX + SCI + CoQ10 4. OVX + SCI + vehicle 5. OVX + Sham	No mention	10 mg/kg/day CoQ10	Post-SCI; treatment start at 1 DPI for 3 weeks after surgery	Motor function Demyelination Tissue damage Inflammation
	Doperalski et al. [57]	Rats, Sprague-Dawley, (267 ± 4 g)	4 ± 0.5 months	36^(c)^	Ovariectomized (1 week prior to SCI)	Hemisection *(C2HS)* incised with a spring scissors and an approximately 1 mm gap was created at the incision site using a pulled micropipette and suction	C2	4 groups^(d)^: 1. SCI + plethysmography 2. OVX + SCI + plethysmography 3. SCI + phrenic motor output 4. OVX + SCI + phrenic motor output	Gas exposition	Plethysmography - exposure to baseline (21% O2, balance N2) or hypercapnic gas for 10 min (7% CO2, 21% O2, and balance N2) Neurophysiology - hypoxia: 4 min at FIO_2_ = 0.12-0.14 - hypercapnia: 5 min at PETCO_2_ ~ 80 mmHg asphyxia: 10-20 sec without ventilation	Post-SCI	Tissue damage Demyelination
	Hubscher et al. [58]	Rats, Wistar	No mention	56	Ovariectomized, 2 weeks pre-SCI	Contusion	T8	4 groups: 1. cycling 2. cycling + SCI 3. OVX 4. OVX + SCI	N.A.	N.A.	Post-SCI (measurement)	Behavioural testing - dorsolateral trunsensitivity in the T5-T7 dermatomes Tissue damage Demyelination
	Ghorbanpoor et al. [59]	Rats, Sprague-Dawley, 200-250 g	adult	96	Intact ovaries	Electrolytic	T8-T9	4 groups: 1. Intact 2. LAM + vehicle 3. STT + vehicle 4. STT + letrozole	Intrathecal injection	1 mg/kg letrozole	Post-SCI; immediately (5 minutes after electrical lesion) and daily for 28 days	Motor function Glial scar formation

^(a)^Information obtained from authors’ previous publication (they referred the reader there for more details about the injury procedure).

^(b)^Total number of animals not stated, an approximate number obtained by sum of numbers reported in visuals’ descriptions.

^(c)^Both values reported.

^(d)^Amount gotten by summing up the numbers reported in groups, but in group numbers speculated that the 2 outcomes (plethysmography and phrenic motor output) weren’t measured in same animals.

### Estrogen, estrogen like compounds and selective estrogen modulators

Estradiol treatment in adult and middle-aged female Sprague-Dawley rats improved recovery of hind-limb locomotion and white matter sparing in both post- and pre-menopausal rats. Pre-injury administration of estradiol also reduced apoptotic cell death in the gray matter in young and old rats. Additionally, decreased body weight gain was observed with estrogen administration in both age groups [38]. In ovariectomized Sprague-Dawley rats with contusive T10 SCI, estradiol treatment led to improved locomotor outcomes, as assessed by the BBB (Basso, Beattie, Bresnahan) score, at 7 days post-injury compared to vehicle-treated controls. However, these differences were no longer significant by day 21 post-injury [39]. In another Sprague-Dawley rat model of T9 compression SCI, estradiol significantly improved locomotor activity, demonstrated by enhanced performance in both the BBB locomotor score and inclined plane test [40]. These functional improvements were accompanied by reduced necrosis at the lesion site. Mechanistically, estradiol suppressed the upregulation of autophagic markers including LC3II, beclin-1, ATG5, and ATG7, while restoring p62 expression, indicating improved regulation of autophagic flux. Estradiol also reduced LC3 expression in motor neurons, suggesting a protective effect by preventing autophagic overactivation and promoting neuronal survival [40]. Estradiol treatment in ovariectomized Wistar rats with compression T10 injury reduced the apoptosis of nerve cells following gradual chronic spinal cord injury [41]. Further supporting its anti-apoptotic action, estradiol was shown to inhibit JNK (c-Jun N-terminal kinase) phosphorylation in ovariectomized Sprague-Dawley rats with a T9–T10 contusion injury, thereby attenuating apoptosis and maintaining neural cell viability [42]. Combination therapies have also been explored. Combining estradiol with dexamethasone, a corticosteroid commonly used in SCI treatment, enhanced functional recovery in Sprague-Dawley rats with T10 contusive injury [43]. This dual treatment decreased the expression of Caspase-3 and IL-6, while upregulating Bcl-2, a key anti-apoptotic factor. Bioinformatics analysis suggested that Bcl-2 is connected to Caspase-3 via CSF3 (colony-stimulating factor 3) and that Caspase-3 is linked to IL-6 through ATF3 (activating transcription factor 3), further supporting the mechanistic role of estradiol in modulating apoptosis and inflammation [43].

Beyond estradiol itself, selective estrogen receptor agonists have shown promise. In a Sprague-Dawley rat model of T9–T11 compression injury, bazedoxifene, a third-generation selective estrogen receptor modulator, significantly suppressed the phosphorylation of MAPKs, specifically p38 and ERK, leading to reduced expression of the pro-inflammatory cytokine IL-6. These molecular effects were associated with a reduction in apoptosis and tissue damage, as well as enhanced remyelination. Functionally, BZA treatment contributed to improved locomotor recovery, suggesting its potential utility in promoting both structural and functional repair following SCI [44]. In adult female C57BL/6 J mice with a T7–T9 crushing injury, the membrane estrogen receptor agonist G-1 mimicked estradiol’s effects. G-1 treatment prevented apoptosis and enhanced motor function, while activating GPR30, which regulated PI3K/Akt and MAPK/ERK signaling pathways. This led to increased expression of anti-apoptotic proteins Bcl-2 and BDNF, and decreased levels of Bax and cleaved caspase-3 [45].

In another model, ovariectomized Sprague-Dawley rats received estradiol-filled or empty implants prior to a moderate contusion at T9–T10. Estradiol-treated animals showed improved locomotor recovery across 7, 14, 21, and 28 days post-injury based on the BBB test. Additionally, both estradiol and tamoxifen treatments promoted white matter sparing during SCI [46]. Using a similar T9–T10 contusion model in Sprague-Dawley rats, another study found that tamoxifen, when administered up to 24 h post-injury, significantly improved locomotor function, preserved white matter, reduced axonal degeneration, and decreased gliosis [47]. Further research investigated the combined effects of tamoxifen and exercise in a T13–L1 hemisection model in Sprague-Dawley rats. All treated groups showed improved hindlimb kinematics, with the combination of tamoxifen and exercise being the most effective intervention. Tamoxifen primarily reduced inflammation and gliosis, while exercise promoted neural plasticity and tissue preservation [48]. However, not all findings have been positive. In C57BL/6 J wild-type mice with a T9 contusion injury, delayed tamoxifen treatment did not improve functional or structural recovery. No significant differences were observed in locomotor performance, tissue sparing, demyelination, glial scar formation, or cell proliferation between tamoxifen-treated and control groups. Behavioral tests revealed no improvement in hindlimb motor function, and histological analyses showed no changes in white matter sparing, axon density, glial reactivity, or myelination [49]. In a thoracic contusion model of SCI in Sprague–Dawley rats, tamoxifen reduced mechanical allodynia in females but not in males [50]. Sex-specific differences were observed in glutamate transporter expression, with females showing higher excitatory amino acid transporter-1 levels and males higher excitatory amino acid transporter-2 levels. Spinal cord injury was associated with altered sensory fiber organization in the dorsal horn, reflected by an increased calcitonin gene-related peptide-α to isolectin B4 ratio compared with sham animals [50]. However, the reduction in allodynia observed in tamoxifen-treated females was not accompanied by changes in glutamate transporter expression or dorsal horn fiber distribution at 28 days post-injury [50].

### Androgen hormones

Dehydroepiandrosterone (DHEA) was applied post-injury in CD-1 mice with a moderate contusive T8 injury [51, 52]. The DHEA-treated group experienced enhanced recovery of left-right coordination and fine motor control compared to vehicle-treated animals. Further, DHEA significantly increased the spared white matter at the injury site while reducing reactive gliosis surrounding the lesion [51]. In a subsequent publication from the same research group, the DHEA-treated mice recovered autonomic bladder function more rapidly (by day four after the injury) than vehicle-treated mice (by day eight post-injury) [51]. In Sprague-Dawley rats with T8 compression injury, the potential of dehydroepiandrosterone sulfate (DHEAS) and pregnenolone (PREG) as part of a combination therapy was investigated alongside the anti-inflammatory agent indomethacin (IM) and the cytokine secretion stimulator bacterial lipopolysaccharide (LPS) [53]. The PREG/LPS/IM combination resulted in a statistically significant improvement in motor function compared to controls. By day 21, four out of six animals in the PREG/LPS/IM group exhibited near-normal walking ability, whereas none of the animals in the other treatment groups achieved a similar level of functional recovery [53]. Lesion sizes in the DHEAS/LPS/IM and PREG/LPS/IM groups were comparable and not statistically different, yet both were significantly smaller than in the other treatment groups. Additionally, cavitation within the lesions was reduced in these two groups, as well as in those receiving LPS alone or LPS/IM. However, cavitation was not significantly diminished in groups treated solely with IM, DHEAS, or PREG, suggesting that LPS may play a crucial role in reducing lesion cavitation [53].

In a study using Sprague-Dawley rats with contusive injury at T9, dihydrotestosterone (DHT) treatment, either alone or combined with estradiol did not yield significant changes in motor recovery (as measured by BBB score) as compared to untreated SCI animals [54]. Sensory function, assessed through voiding behavior, showed significant improvement in groups treated with DHT, estradiol, and their combination. Estradiol treatment resulted in significantly smaller lesion volumes compared to all other groups, including those treated with DHT [54]. Additionally, estradiol-treated animals exhibited smaller percent lesion volumes and larger percentages of spared tissue relative to untreated SCI animals. In contrast, DHT treatment showed no significant differences in these measures compared to untreated SCI animals. Hormone treatment did not affect the number or size of quadriceps motoneuron somata [54]. However, it did attenuate SCI-induced dendritic atrophy. Dendritic lengths in hormone-treated SCI groups were comparable to those of sham animals and significantly longer than in untreated SCI animals. Notably, rostrocaudal dendritic extent remained consistent across all groups [54]. Finally, treatment with DHT, either alone or in combination with estradiol, mitigated SCI-induced muscle fiber atrophy [54]. Another study explored the sexual dimorphism in recovery from SCI, focusing on whether the superior spontaneous recovery in female rats and mice compared to males is linked to the suppressive effect of androgens on T cell-mediated protective responses in the central nervous system [15]. DHT introduced to female Sprague-Dawley rats prior to severe contusive injury had a significantly detrimental effect on functional recovery, as measured by locomotor scores in an open field test. Furthermore, recovery was significantly better in castrated males than in their non-castrated male littermates [15].

### Other interventions

In Sprague-Dawley rats with a T10 contusive SCI, there were no differences in locomotor function(BBB) or spinal cord morphology (across spared tissue and injury length) in animals treated with progesterone as compared to the control group [55]. In a menopausal rat model using ovariectomized Wistar rats, the group receiving both SCI and Coenzyme Q10 treatment showed significantly reduced demyelination compared to the ovariectomized SCI group without treatment. Furthermore, Coenzyme Q10 administration led to a significant reduction in TNF-α levels, along with decreased expression of the pro-apoptotic protein Bax and aquaporin-4 (AQP4) [56]. Respiratory recovery was investigated in a cervical C2 hemisection model comparing ovariectomized, intact female, and male Sprague-Dawley rats. Initially, all groups showed similar disruptions in breathing, reduced ventilation and elevated respiratory frequency. However, during a hypercapnic respiratory challenge, intact females demonstrated a significantly greater increase in ventilation relative to pre-injury levels, unlike males and ovariectomized females. Neurophysiological recordings revealed enhanced phrenic nerve activity in intact females, suggesting an estrogen-dependent improvement in respiratory plasticity following SCI [57]. Pain sensitivity outcomes were explored in another study comparing male, ovariectomized female, and intact cycling female rats following SCI. A significantly lower proportion of intact females developed at-level allodynia compared to both ovariectomized females and males. However, white matter sparing did not differ significantly between cycling and ovariectomized groups, suggesting that hormonal status primarily influenced sensory rather than structural outcomes [58]. Finally, in healthy adult female rats, inhibition of estrogen synthesis via the aromatase inhibitor letrozole led to heightened mechanical allodynia in both hind paws. SCI alone increased reactive microglia and glial fibrillary acidic protein (GFAP)-expressing astrocytes in the dorsal horn, but aromatase inhibition exacerbated these changes, indicating enhanced gliosis. Moreover, the severity of mechanical allodynia positively correlated with gliosis, further supporting a protective role for estrogen in modulating glial responses to injury [59].

## Discussion

This systematic review examined the neuroprotective role of sex hormones in women with SCI and in female animal models of SCI. In human studies, the available evidence was limited and primarily focused on comparisons of cardiovascular risk factors and bone structure between small groups of pre- and postmenopausal women with chronic SCI. Notably, no studies investigated the role of sex hormones in neurological recovery during the early phase post-injury. In contrast, findings from experimental models demonstrated that estradiol consistently exerted broad neuroprotective effects across multiple outcomes. Letrozole, an aromatase inhibitor, was associated with negative effects on motor function, further supporting the protective role of estrogens. Additionally, the presence of intact ovaries was associated with improved sensory function, again suggesting a beneficial role of endogenous estradiol. Evidence on progesterone and DHEA(s) were mixed; however, DHEA appears promising, with two out of three studies reporting positive effects on motor and sensory function as well as tissue preservation.

### Clinical relevance of current evidence

The current review consistently demonstrates the neuroprotective effects of estradiol in female models of SCI. These findings align with previous systematic reviews reporting similar benefits in both male and female animal models [27]. However, human studies have yet to investigate the role of estrogen or estrogen profiles during the early post-injury phase in women with SCI. Insights from traumatic brain injury (TBI) research suggest that hormonal status may influence neurological recovery. For example, menstrual cycle phase at the time of injury or presence of amenorrhea following TBI were linked to poorer recovery outcomes including worse scores on health-related quality of life, global TBI outcomes, and community participation measures [60, 61]. Since the menstrual cycle reflects cumulative exposure to ovarian hormones, its characteristics such as cycle length and regularity may serve as useful proxies for underlying hormonal patterns [62]. Amenorrhea and other disruptions in menstrual regularity may therefore be relevant markers for identifying women at risk of poorer outcomes after neurotrauma. Prolonged estrogen deficiency is also a hallmark of menopause or the menopausal transition, which typically occurs around the age of 51. During this period, aging-related biological processes, combined with the loss of estradiol’s neuroprotective effects and the emergence of a pro-inflammatory state, may exacerbate neurological deficits and contribute to the development of secondary health conditions such as osteoporosis or obesity [63]. To date, only one study has explored the influence of reproductive stage at the time of injury on neurological outcomes in women with SCI. Due to the absence of menopausal status data, the researchers used age as a proxy, comparing outcomes between women presumed to be premenopausal (<40 years) and postmenopausal (>50 years) [22]. No significant differences in Functional Independence Measure (FIM) scores were found. However, this study was further limited by the lack of data on hormone therapy use and medications that can disrupt the hypothalamic–pituitary–gonadal (HPG) axis, such as muscle relaxants, opioid analgesics, and tricyclic antidepressants. Future research should include comparisons between women with and without amenorrhea, or between pre- and postmenopausal women, to better understand the relationship between endogenous estrogen levels and neurological recovery. This is crucial considering demographic shifts in SCI, and an increasing number of women affected by the injury, especially atan older age [64, 65]. Such insights could inform the development of interventional studies to evaluate the effectiveness of estrogen supplementation in the acute and subacute phases of SCI. Importantly, any intervention must consider the potential risks associated with estradiol use following major trauma, including thromboembolic events [66, 67]. One promising avenue could involve selective estrogen receptor modulators (SERMs) such as bazedoxifene or tamoxifen, which have shown benefits in experimental models and may retain estrogenic neuroprotective effects while minimizing adverse outcomes [66].

DHEA and its sulfate ester (DHEAs) are the most abundant steroid hormones in human systemic circulation [68]. Primarily synthesized in the adrenal glands, these hormones can be metabolized into androgens and estrogens, or act directly through multiple mechanisms. Owing to their high baseline levels and the marked decline in production with age, DHEA and DHEAS have been implicated in a wide range of physiological processes related to health and aging [68]. In addition to their peripheral synthesis, DHEA and DHEAS are also produced locally in the brain by neurons and glial cells, classifying them as neurosteroids [69]. Within this context, numerous studies have demonstrated their neuroprotective properties across a variety of experimental models, including those of excitotoxicity, ischemia, neurodegenerative diseases, traumatic brain injury, spinal cord injury, and neuroinflammation [51, 69, 70]. Importantly, the secretion of DHEA declines with age, suggesting that older women who represent a growing proportion of individuals sustaining SCI may have diminished levels of this potentially neuroprotective hormone. This age-related decline in DHEA could contribute to poorer recovery outcomes and warrants further investigation.

### Limitations of current review and underlying evidence

This review identified only two studies investigating the association between hormonal status and health-related outcomes in women with SCI. Both focused on chronic SCI and excluded neurological outcomes. While animal studies offered relatively consistent findings, none fully met all quality criteria outlined in the SYRCLE’s tool for assessing methodological rigor in preclinical research. To address the knowledge gap in women with SCI and reduce heterogeneity, we restricted our review to female human participants and female animal models. Despite this focused approach, considerable variability remained across studies in terms of animal models used, hormone types, dosing regimens, intervention durations, and outcome assessment time points, limiting the generalizability of findings.

First, most studies used adult or young adult animals, with injuries typically at the T8–T10 spinal levels. Contusion (59%) and compression (32%) models were the most frequently employed. Despite no single animal model can fully replicate the complex nature of human SCI, the most commonly used model across the studies- the contusion model- is especially prone to variability and may limit the reproducibility of the findings. The contusion model involves transient forces via weight-drop, electromagnetic, or air-pressure devices and often causes a “weight bounce” phenomenon, where the impactor rod rebounds after the initial strike, leads to inconsistent injury severity, and impact duration is not precisely controlled. In contrast, compression models apply sustained pressure and offer greater consistency, though they reflect different injury mechanisms [71].

Second, outbred animal strains, primarily Sprague-Dawley rats and CD1 mice (77.3%) were commonly used, offering genetic diversity that may better approximate the heterogeneity seen in human populations. This is particularly relevant for immune response variability, which can influence recovery and the effects of immune-modulating treatments [72]. Inbred strains, while more genetically uniform, may not reflect the complexity of human responses to SCI interventions. However, Sprague-Dawley rats are known for lower anxiety levels, which may influence behavioral assays and recovery metrics across studies [73].

Third, several preclinical studies employed ovariectomized female animals to simulate menopause. However, such models involve abrupt hormonal withdrawal, which does not mirror the gradual hormonal decline experienced during the human perimenopausal transition (typically spanning 5–7 years). This limits the translational relevance of findings, especially for women of reproductive age a group that comprises a substantial portion of the female SCI population [74].

Fourth, there was substantial variation in hormone dosages and outcome assessment time points across studies. Although this suggests a potential therapeutic window for multiple sex hormones (including estradiol, estrogenic compounds, and DHEA), the absence of reported human-equivalent doses makes interpretation difficult. Without knowing whether the tested doses align with therapeutic ranges in humans, it is challenging to draw clinically meaningful conclusions.

Lastly, it is important to acknowledge that preclinical research is prone to publication bias, with studies reporting statistically significant or positive effects being more likely to be published [75, 76]. This bias may exaggerate the apparent protective effects of sex hormones observed across animal studies. We did not perform a formal meta-analysis or construct funnel plots due to the heterogeneity of study designs, hormone regimens, and outcome measures; however, readers should interpret the preclinical findings with caution, recognizing that the evidence base may overrepresent positive outcomes.

## Conclusions

Preclinical studies consistently demonstrate that estrogen, estrogenic compounds, and DHEA enhance motor function, reduce tissue damage, and mitigate inflammation and demyelination in female models of SCI. These consistent findings across diverse animal models suggest the presence of conserved neuroprotective mechanisms. However, the translation of these effects into clinical benefits remains uncertain and warrants further investigation.

Considerable variability remained across studies in terms of animal models, hormone types, dosing regimens, intervention durations, and timing of outcome assessments. Future research should aim to clarify the role of both endogenous and exogenous sex hormones in neurological recovery during the acute and subacute phases of SCI in women. Although pre-clinical studies consistently demonstrate neuroprotective effects, these findings have yet to be translated into well-designed clinical trials. Addressing this translational gap is critical for the development of adequately powered randomized controlled trials to evaluate the safety, optimal timing, dosing, and efficacy of hormone-based interventions. Ultimately, such efforts could inform evidence-based guidelines for the use of sex hormones or related compounds in SCI rehabilitation, providing a promising pathway to improve neurorehabilitation strategies for women.

## Supplementary information

Online supplement

## Data Availability

Data sets used in current research are presented within the main text and online supplement.
