# Supplementary material for: The role of sex hormones in neurological and functional recovery following spinal cord injury in females: a systematic review of the literature
Source: Spinal Cord. 2026 Apr 11;64(5):403–15. doi: 10.1038/s41393-026-01201-5 (PMC13658028; doi:10.1038/s41393-026-01201-5)
Supplement: Supplementary file 1 [file 41393_2026_1201_MOESM1_ESM.docx]

**The role of sex hormones in neurological and functional recovery following spinal cord injury in females: a systematic review of the literature**

Ema Vidakovic^1,2^, Elisabeth Bamberg^3^, Björn Zörner^,4^, Kathleen Ostheim^1,2^, Beatrice Minder^5^, Sue Bertschy^1^, Juergen Pannek^1,4^, Petra Stute^6^, Marija Glisic^1,3^

^1^ Swiss Paraplegic Research, Nottwil, Switzerland

^2^ Faculty of Health Sciences and Medicine, University of Lucerne, Lucerne, Switzerland

^3^ Institute of Social and Preventive Medicine, University of Bern, Bern, Switzerland

^4^ Swiss Paraplegic Center, Nottwil, Switzerland

^5^ Public Health & Primary Care Library, University Library of Bern, University of Bern, Switzerland.

^6^ Department of Obstetrics and Gynecology, University Clinic Inselspital Bern, Bern, Switzerland

**Corresponding author:** Prof. Marija Glisic, Mittelstrasse 43, 3012 Bern, Switzerland, email: [marija.glisic@unibe.ch](mailto:marija.glisic@unibe.ch)

**Supplemental table 1.** Methodological Quality assessment of human studies

| **Year** | **Study Design** | **1** | **2** | **3** | **4** | **5** | **6** | **7** | **8** | **9** | **10** | **11** | **12** | **13** | **14** | Overall |
| --- | --- | --- | --- | --- | --- | --- | --- | --- | --- | --- | --- | --- | --- | --- | --- | --- |
| **Hosier et al., 2012** | Cross-sectional | Y | N | Y | Y | N | N | NA | NA | Y | N | Y | NR | NA | N |  |
| Slade et al., 2005 | Case-control | Y | N | N | Y | Y | Y | NA | Y | N | Y | NR | N | --- | --- |  |
| Seijas et al., 2025 | Longitudinal | Y | Y | Y | Y | N | Y | Y | Y | Y | N | Y | NR | Y | Y |  |
| Abbreviations: CD, cannot determine; NA, not applicable; NR, not reported  **Criteria used to asses risk of bias of cohort/cross-sectional studies:**  1. Was the research question or objective in this paper clearly stated?  2. Was the study population clearly specified and defined?  3. Was the participation rate of eligible persons at least 50%?  4. Were all the subjects selected or recruited from the same or similar populations (including the same time period)? Were inclusion and exclusion criteria for being in the study prespecified and applied uniformly to all participants?  5. Was a sample size justification, power description, or variance and effect estimates provided?  6. For the analyses in this paper, were the exposure(s) of interest measured prior to the outcome(s) being measured?  7. Was the timeframe sufficient so that one could reasonably expect to see an association between exposure and outcome if it existed?  8. For exposures that can vary in amount or level, did the study examine different levels of the exposure as related to the outcome (e.g., categories of exposure, or exposure measured as continuous variable)?  9. Were the exposure measures (independent variables) clearly defined, valid, reliable, and implemented consistently across all study participants?  10. Was the exposure(s) assessed more than once over time?  11. Were the outcome measures (dependent variables) clearly defined, valid, reliable, and implemented consistently across all study participants?  12. Were the outcome assessors blinded to the exposure status of participants?  13. Was loss to follow-up after baseline 20% or less?  14. Were key potential confounding variables measured and adjusted statistically for their impact on the relationship between exposure(s) and outcome(s)?  **Criteria used to asses risk of bias of case-control studies:**  1. Was the research question or objective in this paper clearly stated and appropriate?  2. Was the study population clearly specified and defined?  3. Did the authors include a sample size justification?  4. Were controls selected or recruited from the same or similar population that gave rise to the cases (including the same timeframe)?  5. Were the definitions, inclusion and exclusion criteria, algorithms or processes used to identify or select cases and controls valid, reliable, and implemented consistently across all study participants?  6. Were the cases clearly defined and differentiated from controls?  7. If less than 100 percent of eligible cases and/or controls were selected for the study, were the cases and/or controls randomly selected from those eligible?  8. Was there use of concurrent controls?  9. Were the investigators able to confirm that the exposure/risk occurred prior to the development of the condition or event that defined a participant as a case?  10. Were the measures of exposure/risk clearly defined, valid, reliable, and implemented consistently (including the same time period) across all study participants?  11. Were the assessors of exposure/risk blinded to the case or control status of participants?  12. Were key potential confounding variables measured and adjusted statistically in the analyses? If matching was used, did the investigators account for matching during study analysis? | | | | | | | | | | | | | | | | |

**Supplemental table 2.** Methodological quality assessment of animal studies

|  | Chaovipoch, 2006 | Swartz, 2006 | Lin, 2016 | Li, 2007 | Rong, 2012 | Li, 2020 | Kim, 2021 | Cheng, 2016 | Mosquera, 2014 | Colon, 2015 | Ossuna-Carrasco, 2016 | Pukos, 2020 | Fiore, 2003 | Mure, 2004 | Guth, 1994 | Sengelaub, 2018 | Hauben, 2002 | Fee, 2007 | Hassanzadeh, 2018 | Doperalski, 2008 | Hubscher, 2010 | Ghorbanpoor, 2014 | Colon-Mercado, 2024 |
| --- | --- | --- | --- | --- | --- | --- | --- | --- | --- | --- | --- | --- | --- | --- | --- | --- | --- | --- | --- | --- | --- | --- | --- |
| Sequence generation |  |  |  |  |  |  |  |  |  |  |  |  |  |  |  |  |  |  |  |  |  |  |  |
| Baseline characteristics |  |  |  |  |  |  |  |  |  |  |  |  |  |  |  |  |  |  |  |  |  |  |  |
| Allocation concealment |  |  |  |  |  |  |  |  |  |  |  |  |  |  |  |  |  |  |  |  |  |  |  |
| Random housing |  |  |  |  |  |  |  |  |  |  |  |  |  |  |  |  |  |  |  |  |  |  |  |
| Blinding of study personnel |  |  |  |  |  |  |  |  |  |  |  |  |  |  |  |  |  |  |  |  |  |  |  |
| Random outcome assessment |  |  |  |  |  |  |  |  |  |  |  |  |  |  |  |  |  |  |  |  |  |  |  |
| Blinding of outcome assessors |  |  |  |  |  |  |  |  |  |  |  |  |  |  |  |  |  |  |  |  |  |  |  |
| Incomplete outcome data |  |  |  |  |  |  |  |  |  |  |  |  |  |  |  |  |  |  |  |  |  |  |  |
| Selective outcome reporting |  |  |  |  |  |  |  |  |  |  |  |  |  |  |  |  |  |  |  |  |  |  |  |
| Other sources of bias |  |  |  |  |  |  |  |  |  |  |  |  |  |  |  |  |  |  |  |  |  |  |  |

**Appendix 1.** Search strategy used in current systematic review

**Search concepts** (block-building approach according to PICO)

**Embase.com**

| #1 | **P1: Spinal cord injury**  ('spinal cord injury'/exp OR 'cervical spine injury'/de OR 'spinal cord ischemia'/de OR 'paraplegia'/de OR 'spastic paraplegia'/de OR 'quadriplegia'/de OR 'spinal dysraphism'/de OR ('injury'/exp AND 'spinal cord'/exp) OR (((spine OR spinal OR vertebr*) NEAR/3 (trauma* OR injur* OR damag* OR wound*)) OR ('spinal cord' NEAR/3 (disease* OR contusi* OR laceration* OR transection* OR lesion* OR trauma* OR ischemi* OR ischaemi*)) OR (myelopath* NEAR/3 (trauma* OR post-trauma* OR posttrauma*)) OR SCI-group* OR 'central cord injury syndrome*' OR 'central cord syndrome*' OR 'central spinal cord syndrome*' OR 'cauda equine syndrome*' OR 'anterior cord syndrome*' OR 'conus medullaris syndrome*' OR 'Brown Sequard*' OR paraplegi* OR quadriplegi* OR tetraplegi*):ab,ti,kw) | 225,136 |
| --- | --- | --- |
| #2 | **P2: Traumatic brain injury**  ('traumatic brain injury'/de OR 'chronic traumatic encephalopathy'/de OR 'postconcussion syndrome'/de OR ('traumatic brain injur*' OR TBI OR TBIs OR mTBI OR ((brain OR cerebr* OR cerebell*) NEAR/3 (trauma* OR posttrauma* OR lesion* OR injur* OR concussi* OR contusi*)) OR ((traumatic OR posttrauma* OR post-trauma*) NEAR/3 (encephalopath*)) OR ((post-concussi* OR postconcussi* OR subconcussi* OR post-contusi* OR postcontusi* OR post-trauma* OR posttrauma*) NEAR/3 (symptom* OR syndrom* OR encephalopath*))):ab,ti,kw) | 272’185 |
| #3 | #1 OR #2 | 486’622 |
| #4 | **P3: Female population**  ('female'/exp OR 'sex difference'/de OR (female* OR woman* OR women* OR girl* OR gender* OR sexes OR ((sex*) NEAR/3 (differ* OR dimorphism* OR dichromatism* OR role* OR specific* OR depend* OR characteristic*)) OR (biologic* NEAR/3 sex*)):ab,ti,kw) | 14’578’002 |
| #5 | #3 AND #4 | 204’373 |
| #6 | ('estrogen'/de OR 'estradiol derivative'/de OR 'estradiol benzoate'/de OR 'conjugated estrogen'/de OR 'estrogen blood level'/exp OR (estrogen* OR oestrogen*):ab,ti,kw,tn,rn) OR ('estrone'/de OR 'estrone blood level'/de OR (estrone* OR oestrone*):ab,ti,kw,tn,rn) OR ('estradiol'/de OR 'estradiol blood level'/de OR 'estradiol derivative'/de OR (estradiol* OR oestradiol*):ab,ti,kw,tn,rn) OR ('estriol'/de OR 'estriol blood level'/de OR (estriol* OR oestriol*):ab,ti,kw,tn,rn) OR ('testosterone'/de OR 'testosterone blood level'/de OR 'androstanolone'/de OR (testosteron* OR androstanolon* OR dihydrotestosteron* OR dihydroepitestosteron*):ab,ti,kw,tn,rn) OR ('follitropin'/de OR 'follitropin blood level'/de OR ('follicle stimulating hormon*' OR 'folliculostimulating hormon*' OR follitropin* OR follitrophin* OR follitropin* OR folltropin* OR follicotropin* OR FSH OR hFSH):ab,ti,kw,tn,rn) OR ('luteinizing hormone'/de OR 'luteinizing hormone blood level'/de OR ('luteinizing hormon*' OR 'luteinising hormon*' OR lutotropin* OR lutropin* OR 'plasma LH'):ab,ti,kw,tn,rn) OR ('prasterone'/exp OR 'prasterone blood level'/de OR 'prasterone sulfate'/de OR 'prasterone sulfate blood level'/de OR (dihydroepiandrosteron* OR dehydroisoandrosteron* OR dehydroepiandrosterone* OR DHEA OR DHEAs OR prasteron* OR androsten* OR androstenolon*):ab,ti,kw,tn,rn) OR ('progesterone'/de OR 'progesterone blood level'/de OR (progest* OR pregnenedione*):ab,ti,kw,tn,rn) OR ('prolactin'/de OR (prolactin* OR 'lactogenic hormon*' OR mammotropi* OR mammatropi*):ab,ti,kw,tn,rn) OR ('sex hormone'/mj OR ((exogenous NEAR/2 sex NEAR/2 steroid*) OR 'ovarian hormon*' OR (sex* NEAR/2 hormon*) OR 'hormon* supplement*'):ab,ti,kw,tn,rn) | 763’356 |
| #7 | **Reproductive physiology and conditions**  (menstrual cycle, menopause, amenorrhea, PCOS, etc.), which are relevant descriptors in both clinical and preclinical (e.g., estrous cycle models) contexts.  ('menopause and climacterium'/exp OR (menopaus* OR postmenopaus* OR perimenopaus* OR climacteri* OR premenopaus*):ab,ti,kw) OR ('amenorrhea'/de OR (amenorrhea* OR amenorrhoea*):ab,ti,kw) OR ('menstrual cycle'/exp OR (menstrual* OR menstruation OR menorrhea* OR menses OR premenstrual* OR 'fertile period*' OR ((follicular OR luteal OR postovulator* OR preovulator* OR ovulator*) NEAR/3 phase*) OR ((oocyte* OR ovum OR ovar* OR egg*) NEAR/3 (develop* OR growth*)) OR oocytogenesis OR oogenesis OR ootidogenesis OR ovogenesis OR ((ovar* OR ovulation OR reproductive) NEAR/3 (cycle*)) OR luteinization OR 'bleeding pattern*'):ab,ti,kw) OR ('ovary polycystic disease'/de OR (PCOS OR PCOD OR ((polycystic OR poly-cystic OR cystic OR sclerocystic OR micropolycystic) NEAR/2 ovar*) OR (multiple NEAR/2 follic* NEAR/2 cyst*) OR (Stein NEAR/2 Leventhal)):ab,ti,kw) | 504’613 |
| #8 | #6 OR #7 | 1’086’621 |
| #9 | #5 AND #8 | 2’932 |
| #10 | **Limits: excluding conference abstracts, letters, editorials, notes**  #9 NOT ([conference abstract]/lim OR [letter]/lim OR [note]/lim OR [editorial]/lim) | 2’163 |
| #11 | **Limits: excluding reviews, systematic reviews, or meta-analyses**  #10 NOT ('systematic review'/de OR 'review'/de OR 'systematic review (topic)'/de OR 'meta analysis'/exp OR 'meta analysis (topic)'/exp) | 1’795 |
| #12 | **Limit to the adult population** (when analyzing human studies, while still retaining preclinical studies)  #11 NOT (([newborn]/lim OR [infant]/lim OR [child]/lim) NOT ([adult]/lim OR [adolescent]/lim)) | 1’687 |

'spinal cord injury'/exp denotes a subject heading (Emtree): exploded
'cervical spine injury'/de denotes a subject heading: descriptor, not exploded
:ab,ti,kw,tn,rn denotes a word in abstract, title, author keywords, Drug trade name, CAS registry number fields
/lim = limit

**Full Search Strategy per Database**

Date searched: 22 Aug 2025

**Embase.com** (1687 hits)

((('spinal cord injury'/exp OR 'cervical spine injury'/de OR 'spinal cord ischemia'/de OR 'paraplegia'/de OR 'spastic paraplegia'/de OR 'quadriplegia'/de OR 'spinal dysraphism'/de OR ('injury'/exp AND 'spinal cord'/exp) OR (((spine OR spinal OR vertebr*) NEAR/3 (trauma* OR injur* OR damag* OR wound*)) OR ('spinal cord' NEAR/3 (disease* OR contusi* OR laceration* OR transection* OR lesion* OR trauma* OR ischemi* OR ischaemi*)) OR (myelopath* NEAR/3 (trauma* OR post-trauma* OR posttrauma*)) OR SCI-group* OR 'central cord injury syndrome*' OR 'central cord syndrome*' OR 'central spinal cord syndrome*' OR 'cauda equine syndrome*' OR 'anterior cord syndrome*' OR 'conus medullaris syndrome*' OR 'Brown Sequard*' OR paraplegi* OR quadriplegi* OR tetraplegi*):ab,ti,kw) **OR** ('traumatic brain injury'/de OR 'chronic traumatic encephalopathy'/de OR 'postconcussion syndrome'/de OR ('traumatic brain injur*' OR TBI OR TBIs OR mTBI OR ((brain OR cerebr* OR cerebell*) NEAR/3 (trauma* OR posttrauma* OR lesion* OR injur* OR concussi* OR contusi*)) OR ((traumatic OR posttrauma* OR post-trauma*) NEAR/3 (encephalopath*)) OR ((post-concussi* OR postconcussi* OR subconcussi* OR post-contusi* OR postcontusi* OR post-trauma* OR posttrauma*) NEAR/3 (symptom* OR syndrom* OR encephalopath*))):ab,ti,kw)) **AND** ('female'/exp OR 'sex difference'/de OR (female* OR woman* OR women* OR girl* OR gender* OR sexes OR ((sex*) NEAR/3 (differ* OR dimorphism* OR dichromatism* OR role* OR specific* OR depend* OR characteristic*)) OR (biologic* NEAR/3 sex*)):ab,ti,kw)) **AND** (('estrogen'/de OR 'estradiol derivative'/de OR 'estradiol benzoate'/de OR 'conjugated estrogen'/de OR 'estrogen blood level'/exp OR (estrogen* OR oestrogen*):ab,ti,kw,tn,rn) OR ('estrone'/de OR 'estrone blood level'/de OR (estrone* OR oestrone*):ab,ti,kw,tn,rn) OR ('estradiol'/de OR 'estradiol blood level'/de OR 'estradiol derivative'/de OR (estradiol* OR oestradiol*):ab,ti,kw,tn,rn) OR ('estriol'/de OR 'estriol blood level'/de OR (estriol* OR oestriol*):ab,ti,kw,tn,rn) OR ('testosterone'/de OR 'testosterone blood level'/de OR 'androstanolone'/de OR (testosteron* OR androstanolon* OR dihydrotestosteron* OR dihydroepitestosteron*):ab,ti,kw,tn,rn) OR ('follitropin'/de OR 'follitropin blood level'/de OR ('follicle stimulating hormon*' OR 'folliculostimulating hormon*' OR follitropin* OR follitrophin* OR follitropin* OR folltropin* OR follicotropin* OR FSH OR hFSH):ab,ti,kw,tn,rn) OR ('luteinizing hormone'/de OR 'luteinizing hormone blood level'/de OR ('luteinizing hormon*' OR 'luteinising hormon*' OR lutotropin* OR lutropin* OR 'plasma LH'):ab,ti,kw,tn,rn) OR ('prasterone'/exp OR 'prasterone blood level'/de OR 'prasterone sulfate'/de OR 'prasterone sulfate blood level'/de OR (dihydroepiandrosteron* OR dehydroisoandrosteron* OR dehydroepiandrosterone* OR DHEA OR DHEAs OR prasteron* OR androsten* OR androstenolon*):ab,ti,kw,tn,rn) OR ('progesterone'/de OR 'progesterone blood level'/de OR (progest* OR pregnenedione*):ab,ti,kw,tn,rn) OR ('prolactin'/de OR (prolactin* OR 'lactogenic hormon*' OR mammotropi* OR mammatropi*):ab,ti,kw,tn,rn) OR ('sex hormone'/mj OR ((exogenous NEAR/2 sex NEAR/2 steroid*) OR 'ovarian hormon*' OR (sex* NEAR/2 hormon*) OR 'hormon* supplement*'):ab,ti,kw,tn,rn) **OR** ('menopause and climacterium'/exp OR (menopaus* OR postmenopaus* OR perimenopaus* OR climacteri* OR premenopaus*):ab,ti,kw) OR ('amenorrhea'/de OR (amenorrhea* OR amenorrhoea*):ab,ti,kw) OR ('menstrual cycle'/exp OR (menstrual* OR menstruation OR menorrhea* OR menses OR premenstrual* OR 'fertile period*' OR ((follicular OR luteal OR postovulator* OR preovulator* OR ovulator*) NEAR/3 phase*) OR ((oocyte* OR ovum OR ovar* OR egg*) NEAR/3 (develop* OR growth*)) OR oocytogenesis OR oogenesis OR ootidogenesis OR ovogenesis OR ((ovar* OR ovulation OR reproductive) NEAR/3 (cycle*)) OR luteinization OR 'bleeding pattern*'):ab,ti,kw) OR ('ovary polycystic disease'/de OR (PCOS OR PCOD OR ((polycystic OR poly-cystic OR cystic OR sclerocystic OR micropolycystic) NEAR/2 ovar*) OR (multiple NEAR/2 follic* NEAR/2 cyst*) OR (Stein NEAR/2 Leventhal)):ab,ti,kw)) **NOT** ([Conference Abstract]/lim OR [Letter]/lim OR [Note]/lim OR [Editorial]/lim) **NOT** ('systematic review'/de OR 'review'/de OR 'systematic review (topic)'/de OR 'meta analysis'/exp OR 'meta analysis (topic)'/exp) **NOT** (([newborn]/lim OR [infant]/lim OR [child]/lim) NOT ([adult]/lim OR [adolescent]/lim))

**Medline** via Ovid (1072 hits)
Ovid MEDLINE(R) ALL <1946 to August 21, 2025>

(((exp spinal cord injuries/ OR exp spinal cord ischemia/ OR exp paraplegia/ OR quadriplegia/ OR spinal dysraphism/ OR ("Wounds and Injuries"/ AND spinal cord/) OR (((spine OR spinal OR vertebr*) ADJ3 (trauma* OR injur* OR damag* OR wound*)) OR (spinal cord ADJ3 (disease* OR contusi* OR laceration* OR transection* OR lesion* OR trauma* OR ischemi* OR ischaemi*)) OR (myelopath* ADJ3 (trauma* OR post-trauma* OR posttrauma*)) OR SCI-group* OR central cord injury syndrome* OR central cord syndrome* OR central spinal cord syndrome* OR cauda equine syndrome* OR anterior cord syndrome* OR conus medullaris syndrome* OR Brown Sequard* OR paraplegi* OR quadriplegi* OR tetraplegi*).ab,ti,kf.) **OR** (exp Brain Injuries, Traumatic/ OR chronic traumatic encephalopathy/ OR post-concussion syndrome/ OR (traumatic brain injur* OR TBI OR mTBI OR TBIs OR ((brain OR cerebr* OR cerebell*) ADJ3 (trauma* OR posttrauma* OR lesion* OR injur* OR concussi* OR contusi*)) OR ((traumatic OR posttrauma* OR post-trauma*) ADJ3 (encephalopath*)) OR ((post-concussi* OR postconcussi* OR subconcussi* OR post-contusi* OR postcontusi* OR post-trauma* OR posttrauma*) ADJ3 (symptom* OR syndrom* OR encephalopath*))).ab,ti,kf.)) **AND** (female/ OR sex characteristics/ OR (female* OR woman* OR women* OR girl* OR gender* OR sexes OR ((sex*) ADJ3 (differ* OR dimorphism* OR dichromatism* OR role* OR specific* OR depend* OR characteristic*)) OR (biologic* ADJ3 sex*)).ab,ti,kf,rn,ot.)) **AND** ((estrogens/ OR "Estrogens, Conjugated (USP)"/ OR exp Estrogens/bl OR (estrogen* OR oestrogen*).ab,ti,kf,rn,ot.) OR (exp estrone/ OR (estrone* OR oestrone*).ab,ti,kf,rn,ot.) OR (estradiol/ OR exp estradiol/bl OR (estradiol* OR oestradiol*).ab,ti,kf,rn,ot.) OR (estriol/ OR (estriol* OR oestriol*).ab,ti,kf,rn,ot.) OR (testosterone/ OR exp testosterone/bl OR dihydrotestosterone/ OR (testosteron* OR androstanolon* OR dihydrotestosteron* OR dihydroepitestosteron*).ab,ti,kf,rn,ot.) OR (exp Follicle Stimulating Hormone/ OR (follicle stimulating hormon* OR folliculostimulating hormon* OR follitropin* OR follitrophin* OR follitropin* OR folltropin* OR follicotropin* OR FSH OR hFSH).ab,ti,kf,rn,ot.) OR (luteinizing hormone/ OR luteinizing hormone blood level/ OR (luteinizing hormon* OR luteinising hormon* OR lutotropin* OR lutropin* OR plasma LH).ab,ti,kf,rn,ot) OR (exp dehydroepiandrosterone/ OR (dihydroepiandrosteron* OR dehydroisoandrosteron* OR dehydroepiandrosterone* OR DHEA OR DHEAs OR prasteron* OR androsten* OR androstenolon*).ab,ti,kf,rn,ot) OR (exp progesterone/ OR (progest* OR pregnenedione*).ab,ti,kf,rn,ot.) OR (prolactin/ OR (prolactin* OR lactogenic hormon* OR mammotropi* OR mammatropi*).ab,ti,kf,rn,ot) OR (*Gonadal Steroid Hormones/ OR ((exogenous ADJ2 sex ADJ2 steroid*) OR ovarian hormon* OR (sex* ADJ2 hormon*) OR hormon* supplement*).ab,ti,kf,rn,ot) **OR** (exp menopause/ OR (menopaus* OR postmenopaus* OR perimenopaus* OR climacteri* OR premenopaus*).ab,ti,kf.) OR (amenorrhea/ OR (amenorrhea* OR amenorrhoea*).ab,ti,kf.) OR (exp menstrual cycle/ OR (menstrual* OR menstruation OR menorrhea* OR menses OR premenstrual* OR fertile period* OR ((follicular OR luteal OR postovulator* OR preovulator* OR ovulator*) ADJ3 phase*) OR ((oocyte* OR ovum OR ovar* OR egg*) ADJ3 (develop* OR growth*)) OR oocytogenesis OR oogenesis OR ootidogenesis OR ovogenesis OR ((ovar* OR ovulation OR reproductive) ADJ3 (cycle*)) OR luteinization OR bleeding pattern*).ab,ti,kf.) OR (Polycystic Ovary Syndrome/ OR (PCOS OR PCOD OR ((polycystic OR poly-cystic OR sclerocystic OR cystic OR micropolycystic) ADJ2 ovar*) OR (multiple ADJ2 follic* ADJ2 cyst*) OR (Stein ADJ2 Leventhal)).ab,ti,kf.)) **NOT** (letter OR news OR comment OR editorial OR congress).pt. **NOT** (systematic review/ OR review/ OR systematic review as topic/ OR meta analysis/ OR meta-analysis as topic/ OR network meta-analysis/) **NOT** ((exp infant/ OR exp child/) NOT (exp adult/ OR adolescent/ OR universities/))

exp spinal cord injuries/ denotes a Medical Subject Heading (MeSH) term (‘exploded’, i.e., includes all narrower terms)
quadriplegia/ denotes a Medical Subject Heading (MeSH) term (not ‘exploded’: only the term itself)
ab,ti,kf,rn,ot. Restricts the search to words in the abstract, title, author keywords, Registry Number/Name of Substance, or Original Title
systematic review/ before 2005 this was searched as a publication type: systematic review.pt. After the 2005 reload of MEDLINE, publication types were also integrated into MeSH; thus, systematic review/ yields the same results.

**Cochrane CENTRAL** (218 Trials)
Cochrane Central Register of Controlled Trials, Issue 7 of 12, July 2025

#1	(((spine OR spinal OR vertebr*) NEAR/3 (trauma* OR injur* OR damag* OR wound*)) OR ("spinal cord" NEAR/3 (disease* OR contusi* OR laceration* OR transection* OR lesion* OR trauma* OR ischemi* OR ischaemi*)) OR (myelopath* NEAR/3 (trauma* OR post-trauma* OR posttrauma*)) OR (SCI-group*) OR "central cord injury syndrome" OR "central cord syndrome" OR "central spinal cord syndrome" OR "cauda equine syndrome" OR "anterior cord syndrome" OR "conus medullaris syndrome" OR "Brown Sequard" OR paraplegi* OR quadriplegi* OR tetraplegi*)

#2	(traumatic-brain-injur* OR TBI OR TBIs OR mTBI OR ((brain OR cerebr* OR cerebell*) NEAR/3 (trauma* OR posttrauma* OR lesion* OR injur* OR concussi* OR contusi*)) OR ((traumatic OR posttrauma* OR post-trauma*) NEAR/3 (encephalopath*)) OR ((post-concussi* OR postconcussi* OR subconcussi* OR post-contusi* OR postcontusi* OR post-trauma* OR posttrauma*) NEAR/3 (symptom* OR syndrom* OR encephalopath*)))

#3	#1 OR #2

#4	(female* OR woman* OR women* OR girl* OR gender* OR sexes OR ((sex*) NEAR/3 (differ* OR dimorphism* OR dichromatism* OR role* OR specific* OR depend* OR characteristic*)) OR (biologic* NEAR/3 sex*))

#5	#3 AND #4

#6	(estrogen* OR oestrogen* OR estrone* OR oestrone* OR estradiol* OR oestradiol* OR estriol* OR oestriol* OR testosteron* OR androstanolon* OR dihydrotestosteron* OR dihydroepitestosteron* OR follicle-stimulating-hormon* OR folliculostimulating-hormon* OR follitropin* OR follitrophin* OR follitropin* OR folltropin* OR follicotropin* OR FSH OR hFSH OR luteinizing-hormon* OR luteinising-hormon* OR lutotropin* OR lutropin* OR plasma-LH OR dihydroepiandrosteron* OR dehydroisoandrosteron* OR dehydroepiandrosterone* OR DHEA OR DHEAs OR prasteron* OR androsten* OR androstenolon* OR progest* OR pregnenedione* OR prolactin* OR lactogenic-hormon* OR mammotropi* OR mammatropi* OR (exogenous NEAR/2 sex NEAR/2 steroid*) OR ovarian-hormon* OR (sex* NEAR/2 hormon*) OR (hormon* NEXT supplement*))

#7	(menopaus* OR postmenopaus* OR perimenopaus* OR climacteri* OR premenopaus* OR amenorrhea* OR amenorrhoea* OR menstrual* OR menstruation OR menorrhea* OR menses OR premenstrual* OR (fertile NEXT period*) OR ((follicular OR luteal OR postovulator* OR preovulator* OR ovulator*) NEAR/3 (phase*)) OR ((oocyte* OR ovum OR ovar* OR egg*) NEAR/3 (develop* OR growth*)) OR oocytogenesis OR oogenesis OR ootidogenesis OR ovogenesis OR ((ovar* OR ovulation OR reproductive) NEAR/3 (cycle*)) OR luteinization OR bleeding-pattern* OR PCOS OR PCOD OR ((polycystic OR poly-cystic OR cystic OR sclerocystic OR micropolycystic) NEAR/2 ovar*) OR (multiple NEAR/2 follic* NEAR/2 cyst*) OR (Stein NEAR/2 Leventhal))

#8	#6 OR #7

#9	#5 AND #8

***Web of Science Core Collection** (via Clarivate) (1516 hits)

Query link: (building of the query analogous to that of Cochrane CENTRAL)
<https://www.webofscience.com/wos/woscc/summary/ef7246b8-6af6-4459-b1dc-7c9ed59ad949-9cd2b72f/relevance/1>

TS=(((spine OR spinal OR vertebr*) NEAR/3 (trauma* OR injur* OR damag* OR wound*)) OR ("spinal cord" NEAR/3 (disease* OR contusi* OR laceration* OR transection* OR lesion* OR trauma* OR ischemi* OR ischaemi*)) OR (myelopath* NEAR/3 (trauma* OR post-trauma* OR posttrauma*)) OR (SCI-group*) OR "central cord injury syndrome*" OR "central cord syndrome*" OR "central spinal cord syndrome*" OR "cauda equine syndrome*" OR "anterior cord syndrome*" OR "conus medullaris syndrome*" OR "Brown Sequard*" OR paraplegi* OR quadriplegi* OR tetraplegi*)

**OR**

TS=("traumatic brain injur*" OR TBI OR TBIs OR mTBI OR ((brain OR cerebr* OR cerebell*) NEAR/3 (trauma* OR posttrauma* OR lesion* OR injur* OR concussi* OR contusi*)) OR ((traumatic OR posttrauma* OR post-trauma*) NEAR/3 (encephalopath*)) OR ((post-concussi* OR postconcussi* OR subconcussi* OR post-contusi* OR postcontusi* OR post-trauma* OR posttrauma*) NEAR/3 (symptom* OR syndrom* OR encephalopath*)))

**AND**

TS=(female* OR woman* OR women* OR girl* OR gender* OR sexes OR ((sex*) NEAR/3 (differ* OR dimorphism* OR dichromatism* OR role* OR specific* OR depend* OR characteristic*)) OR (biologic* NEAR/3 sex*))

**AND**

TS=(estrogen* OR oestrogen* OR estrone* OR oestrone* OR estradiol* OR oestradiol* OR estriol* OR oestriol* OR testosteron* OR androstanolon* OR dihydrotestosteron* OR dihydroepitestosteron* OR "follicle stimulating hormon*" OR "folliculostimulating hormon*" OR follitropin* OR follitrophin* OR follitropin* OR folltropin* OR follicotropin* OR FSH OR hFSH OR "luteinizing hormon*" OR "luteinising hormon*" OR lutotropin* OR lutropin* OR plasma-LH OR dihydroepiandrosteron* OR dehydroisoandrosteron* OR dehydroepiandrosterone* OR DHEA OR DHEAs OR prasteron* OR androsten* OR androstenolon* OR progest* OR pregnenedione* OR prolactin* OR "lactogenic hormon*" OR mammotropi* OR mammatropi* OR (exogenous NEAR/2 sex NEAR/2 steroid*) OR "ovarian hormon*" OR (sex* NEAR/2 hormon*) OR "hormon* supplement*")

**OR**

TS=(menopaus* OR postmenopaus* OR perimenopaus* OR climacteri* OR premenopaus* OR amenorrhea* OR amenorrhoea* OR menstrual* OR menstruation OR menorrhea* OR menses OR premenstrual* OR "fertile period*" OR ((follicular OR luteal OR postovulator* OR preovulator* OR ovulator*) NEAR/3 (phase*)) OR ((oocyte* OR ovum OR ovar* OR egg*) NEAR/3 (develop* OR growth*)) OR oocytogenesis OR oogenesis OR ootidogenesis OR ovogenesis OR ((ovar* OR ovulation OR reproductive) NEAR/3 (cycle*)) OR luteinization OR "bleeding pattern*" OR PCOS OR PCOD OR ((polycystic OR poly-cystic OR cystic OR sclerocystic OR micropolycystic) NEAR/2 ovar*) OR (multiple NEAR/2 follic* NEAR/2 cyst*) OR (Stein NEAR/2 Leventhal))

AND DT=(article OR early access)

*Science Citation Index Expanded (1900-present) ; Social Sciences Citation Index (1900-present) ; Arts & Humanities Citation Index (1975-present) ; Conference Proceedings Citation Index- Science (1990-present) ; Conference Proceedings Citation Index- Social Science & Humanities (1990-present) ; Emerging Sources Citation Index (2018-present)
